# A Data Envelopment Analysis of the Impact of European Funds on Environmental Indicators

**DOI:** 10.3390/ijerph18062800

**Published:** 2021-03-10

**Authors:** Marcos Ferasso, Miguel Blanco, Lydia Bares

**Affiliations:** 1 Institute of Scientific Research and Graduate School, Universidad de Lima, Av. Javier Prado Este, 4600, Santiago de Surco, Lima 15023, Peru; admmarcosferasso@gmail.com; 2 Department of General Economics, University of Cadiz, Avenue Enrique Villegas Velez, 2, 11002 Cádiz, Spain; miguel.blanco@uca.es

**Keywords:** renewable energy, European Funds, Data Envelopment Analysis (DEA), sustainability

## Abstract

The European Union (EU) has launched two regional investment programs of European Funds (FE) in the last decade. One covers the period of 2007 to 2013, and the second from 2014 to 2020. Among the goals contained in FE regulations is that of achieving sustainable growth through the conversion of fossil energy production systems to renewable energy. This research has had a goal to determine whether the countries of the Eurozone maintain homogeneous levels of efficiency in the use of these resources to improve the levels of environmental quality related to the use of this type of energy. The adopted research method for efficiency analyses was Data Envelopment Analysis (DEA). Findings revealed that the efficiency in the use of renewable energies is very uneven among the analyzed countries and that these differences are maintained throughout the analyzed period. These results suggest that the criteria for the distribution of the funds should be modified. The current distribution is mainly based on the per capita income of the countries and/or regions. In this way, compliance with the European Green Pact approved in September 2020 would be guaranteed.

## 1. Introduction

The Sustainable Development Strategy has become a basic worldview for the world community thanks to the active role of the United Nations (UN) in formulating strategies. The first strategy is described in the Brundtland Commission Report [1] related to the adoption of the 2030 action plan, setting out renewed global sustainable development goals [2]. This report brought to light the latest strategic documents of the European Union (EU), namely the strategy Europe 2020 [3].

In 2010, the EU adopted a 10-year economic development strategy, as described in the Europe 2020: Strategy for Reasonable, Sustainable and Inclusive Growth report [4]. The strategy was developed as a continuation of the Lisbon Strategy 2000–2010 plan [5] as the provisions of the latter were not implemented in the prescribed period. The key idea of the strategy is to coordinate economic growth, protection and preservation of the environment, and social protection of all members of society [6]. Then, human development depends on how to shift from a brown economy to a green one through green strategies development [7]. According to calculations made available by the European Commission (EC), if the goal of generating 20% of energy from renewable sources were reached, more than 600,000 jobs could be created in the EU.

The achievement of a reasonable, sustainable, and integrative growth, of a climate-neutral continent, of developing sustainable solutions (as part of the Strategy Europe 2020 and the updated Annual Sustainable Growth Strategy 2020 plan) involves an integrated approach and means that at the core of these goals will be an industrial strategy with strong foundations in the single market. This strategy enables businesses to innovate and to develop new technologies while boosting circularity and creating new markets and new business opportunities [8]. That means refocusing Europe’s economic policy toward the long term, aiming the offer of a sustainable and prosperous future to younger generations in all parts of Europe [9]. Thus, one of the most relevant trends in EU internal and external policy is the development of environmental policies in order to preserve and restore the European natural environment.

These aforementioned EU strategic goals were outlined in the 7th Environment Action Programme of the EU 2014–2020. These goals can only be achieved if several actions of key international environmental agreements are actively supported and properly implemented, both at the Union level and worldwide. Most environmental problems have a transboundary nature and often a global scope, and they can only be addressed effectively through international cooperation. For this reason, the Lisbon Treaty [10] established that one of the key goals of the EU policy related to the environment is to promote measures at the international level, to deal with regional or worldwide environmental problems, and to combat climate change [11]. The EU takes an active part in the elaboration, ratification, and implementation of multilateral environmental agreements [12].

In an effort to fulfill EU member states’ commitments in the field of climate change and sustainable development, the European Commission approved in March 2018 the Financing Action Plan for Sustainable Development [13]. The situation in the field of climate change and the depletion of natural resources on the planet continues to deteriorate, which necessitates the instance of urgent action to move toward sustainable development [11]. Large-scale tasks are necessary for the implementation of the SDGs, and these require significant additional financial resources.

Therefore, in 2016, the European Commission established a high-level experts group on sustainable financing. In January 2018, this group published a report [14], on the basis of which the Sustainable Finance: Commission’s Action Plan for a Greener and Cleaner Economy [15] report was developed, which complies with the principles of sustainable development.

The transformation of the European economy into a more environmentally friendly, more sustainable, and more circular system will not only help to reduce the EU’s environmental footprint on the planet but also eliminate existing inequalities. According to the Commission’s Action Plan, this will also increase the competitiveness of the EU’s economy by improving the efficiency of production processes and by reducing the cost of access to resources management.

EU adopted financial instruments in order to finance the change regarding the growth model, such as rural development funds (structural funds) derived from the Framework Program for R&D, Trans-European Networks, and the European Investment Bank. Specific studies on territorial development in Europe showed relevant differences in their growth models. Therefore, the distribution of the funds as instruments that make possible the changes toward a sustainable growth model should specifically consider the economic reality of each of the countries.

Thus, the distribution of public resources among countries should take into account their singularities. Otherwise, the process of regional differentiation could generate unbalanced actions. Some countries can present higher levels of economic and environmental growth to the detriment of others, which increases the distance from the initial quantitative goals.

For this reason, it is necessary to carry out an analysis that can relate, at the territorial level, the changes in growth models with the use of the resources received for it. Therefore, the goal of this research is to determine the level of efficiency that the Eurozone countries are achieving in the use of the funds to improve the use of renewable energies.

The contributions of this research are presented. Our results can be used for public administrations to develop regulations, incorporating the degree of environmental efficiency achieved as another weighting parameter for the approval of projects financed with European Funds. Second, the active persons, those presenting the projects, could access a national and territorial database created for this purpose, in which they can find out the specificities of the projects with the best qualification and where they have been implanted.

The following structure has been followed in this manuscript. After this introduction, the main EU funds available to the countries are described. Subsequently, an efficiency analysis model is presented, and the variables to be used are defined. Subsequently, the model is applied to the variables, which allowed a series of conclusions to be drawn, and the limitations of this study are established.

## 2. The European Funds as Financial Instruments Devoted to Finance the Growth Model Transition

The big concern on the part of the EU in improving environmental quality in recent decades caused an important political action aiming at environment preservation [16]. The key source of financial resources to support these actions and improve environmental quality in the EU comes from the European Funds. Among funds, main contributions came from the European Regional Development Funds (ERDF), European Social Fund (ESF), Cohesion Fund (CF), European Agricultural Fund for Rural Development (EAFRD), European Fisheries Fund (EFF), and European Maritime and Fisheries Fund (FEMP).

In the past decades, two funding packages have been approved. The first had a temporal dimension that spanned the period of 2007 to 2013, and the second was from 2014 to 2020. Their regulations included the need to finance projects related to the conservation of the environment. According to the Regulation (EC) No. 1080/2006 of the European Parliament and of the Council of 5 July 2006, the European Regional Development Fund (ERDF) had six environmental measures during the period of 2007–2013, covering topics since the rehabilitation of physical spaces, cleaner transportation, to energy efficiency and using renewable energies.

In the period of 2014–2020, there were 15 ERDF environmental measures included in Regulation (EU) No. 1301/2013 of the European Parliament and of the Council of 17 December 2013. Among these measures, there were increasing considerations of energy efficiency, reducing carbon emissions, investing in low-carbon research and development (R&D), promoting the water and waste sectors, and climate change issues.

In Regulation (EC) No. 1081/2006 of the European Parliament and of the Council of 5 July 2006, there was only one ESF environmental measure relating to improving the adaptability of workers, companies, and entrepreneurs to economic change. Among these measures, it is identified the permanent training in environmentally friendly technologies for the period of 2007–2013. The ESF environmental measure of the period of 2014–2020 was to support the shift to a low-carbon economy adapted to climate change. This shift makes efficient use of resources and is environmentally sustainable by improving the education and training systems required for the adaptation of necessary skills and qualifications. As a result, the improvement of professional skills and the creation of new jobs in sectors related to the environment and energy are expected, according to Regulation (EU) No. 1304/2013 of the European Parliament and of the Council of 17 December 2013.

The Cohesion Fund had two environmental goals during 2007–2013: to adapt the use of these funds to the community policy for the protection of the environment and to facilitate the implementation of policies that present clear benefits for the environment. These policies cover aspects such as energy efficiency and renewable energies, and since transportation is not part of the trans-European networks, rail, river, and maritime transportation, systems of intermodal transportation, and their interoperability. Moreover, these policies focusing on the management of maritime, air and road traffic, clean urban transport, and public transport (Regulation (EC) No. 1084/2006 of the Council of 11 July 2006). However, according to Regulation No. 1300/2013 of the EU of 17 December 2013, for the period of 2014–2020, the Cohesion Funds (CF) included 14 specific actions related to environmental measures, expanding the scope of the previous regulation.

The European Agricultural Fund for Rural Development (EAFRD), in the period of 2007–2013, had four environmental measures according to Council Regulation (CE) No. 1698/2005 of 20 September 2005. In the period of 2014–2020, the EAFRD expanded the focus to eight environmental measures, as stated by Council Regulation No. 1305/2013 of the Council of 17 December 2013.

Finally, the European Fisheries Fund (EFF) and European Maritime and Fisheries Fund (EMFF) presented only one environmental measure in the period of 2007–2013 related to support measures and initiatives aimed at diversifying and promoting economic development in areas affected by the decline in fishing activities, as claimed by Council Regulation EC No. 1198/2006 of 27 July 2006. In the period of 2014–2020, according to EU Regulation No. 508/2014 of 15 May 2014, the measures increase to nine actions toward maritime and fishing activities.

In summary, the regulations governing the funds for the 2014–2020 period are more sensitive to the need to finance projects that consider the environmental dimension. However, it is needed to quantify the efficiency of these resources to ensure sustainable growth.

## 3. Materials and Methods

Efficiency analysis in environmental issues is quite frequent [17,18,19,20,21]. Efficiency is a concept that is traditionally defined as the ability of organizations to produce the maximum with the minimum possible inputs [22]. The inputs in this research are the European Funds used, and the outputs are a series of environmental, economic growth, and employment indicators that show the level of impact that the European programs approved and financed from European Funds. Therefore, the analysis is oriented toward the establishment of a comparison of technical efficiency achieved among the Eurozone’s countries.

For the efficiency analysis, a comprehensive bibliographic search was carried out to detect which methodology had been widely used. The result was that there is a significant number of publications that use the frontier model. In this model, a function of production profit, or cost determined by means of parametric or non-parametric techniques, is defined. Likewise, a production frontier is defined and determines the maximum product that can be achieved from a given combination of inputs [23]. Thus, the greater or lesser efficiency of an organization [24] could be determined by its distance from this level.

A series of units called decision making units (DMUs) were defined, which were originally companies’ departments. Later, a series of inputs and outputs were defined, and a comparative analysis was established by determining which of them were achieving higher levels of efficiency. Depending on the results, corrective measures could be taken on ineffective procedures.

In recent decades, the scope in applying these models has been considerably expanded to the field of public administration, and currently, their application is being frequently used in the assessment of public policies [25].

Therefore, in the construction of the production function, a series of inputs—the European Funds—and a series of outputs—the levels of environmental well-being, economic growth, and employment generated in the renewable energy sector—have been considered. Likewise, the decision making units (DMUs) have been defined. In this research, DMUs are considered as the Eurozone’s countries, motivated by the fact that including in the Eurogroup ensures that their economies move around certain economic levels. Therefore, the countries that pertain to Eurozone and that were analyzed are Germany, Austria, Belgium, Cyprus, Slovakia, Slovenia, Spain, Estonia, Finland, France, Greece, Ireland, Italy, Latvia, Lithuania, Luxembourg, Malta, the Netherlands, and Portugal. All of them, in order to form part of the single currency, had to orient their economic policies toward achieving four basic goals related to (a) price stability, (b) the existence of sound public accounts, (c) stability in their exchange rates, and (d) certain types of long-term interest fixed.

Currently, for maintaining the countries in this area of economic and social influence, it is required that public deficits remain around reference levels. Likewise, monetary policy becomes dependent on a supranational body, such as the European Central Bank. This organization will be in charge of defining the basic market interest rates and adjusting the liquidity of the system according to the evolution of inflation.

Therefore, from an academic point of view, the Eurozone has been consolidating itself as a subject of an economic and social unit of analysis [26,27,28]. In our study, this means leaving out countries such as the United Kingdom (U.K.) that have contributed a significant percentage of the gross domestic product (GDP) of the EU until 2021, the date in which it ceased to be part of it—also known as Brexit. However, the U.K. has not been part of the Eurogroup. Therefore, British monetary and budgetary policies are considered as outside the competence framework of the Eurozone. These types of policies, especially since 2008, have been increasingly considered as an interventionist in the economies of nations to guarantee certain goals of growth and employment. Therefore, its inclusion would mean, for this study, considering a group of countries with common budgetary and monetary policies and others, as is the case of the U.K., with fiscal policies (decided by their political and administrative bodies), and monetary (decided by the Bank of England).

Likewise, a literature search has been carried out to determine parametric or non-parametric models for determining efficiency levels. The result of these investigations has made it possible to determine that the non-parametric Data Envelopment Analysis (DEA) methodology is a research instrument widely used today and that it is frequently applied to various industries [25]. Some examples of using the DEA methodology came from Pardo, Martínez, and Silveira [29] examined energy use, energy efficiency, and CO2 emissions using Data Envelopment Analysis (DEA) from Charnes, Cooper and Rhodes [30] and panel data techniques in 19 subsectors in the Swedish service sectors; Bian, He, and Xu [7] assessing regional energy efficiency in China; and Ebrahimi and Salehi [20] when analyzing the Isfahan province of Iran using a DEA model of Charnes, Cooper and Rhodes [30].

Other applications of DEA methodology can be seen in the studies of Lin and Du [11] when focusing on China’s regional energy and CO_2_ emissions performance; Song, Hao, and Zhu [31], assessing the changes in the environmental efficiency of the Chinese transportation sector; Suzuki, Nijkamp, and Rietveld [32], using an adjusted DEA model for assessing the energy-environment sector in Japan; and Duan, Guo and Xie [33] when measuring energy and CO_2_ emissions performance of thermal power industries in China.

Advancing in the successful applications of DEA methodology, Iftikhar, He, and Wang [34] studied the energy efficiency and CO_2_ emissions for the main economies; Suzuki and Nijkamp [35] compared the evaluation of the efficiency of the energy-environmental-economic objectives for the countries of the EU, APEC, and ASEAN (A&A); Tian, Zhao, Mu, Kanianska, and Feng [36] analyzed the environmental efficiency of China’s open field grape production under the restriction of carbon emissions; and Zha, Zhao, and Bian [37] evaluated the regional efficiency of energy use and CO_2_ emissions in China.

Lastly, successful results obtained through the DEA methodology implementation are seen in the studies of Chen and Geng [38] when using the non-radial Malmquist Indices; Saglam [39], addressing the most efficient renewable energy source; Tang, You, Sun, and Zhang [40] when proposing a parallel measurement model in the Chinese transportation sector; serving as the basis for the emergence of the technical efficiency index [41]. Subsequently, Banker, Charnes, and Cooper [42] developed a model that is known by the initials of its authors (BCC), similar to the previous one, but that includes a more flexible border.

The fact of being able to include multiple input and output variables in this BCC model has made it an efficiency measurement tool usually used in the assessment of policies from the public sector [43], since public organizations pursue multiple goals, due to the difficulty in determining their performance levels [44].

In this model, the efficiency of the DMU is obtained as Equation (1):

(1) $$Ef=\frac{Y}{X}=\frac{\mathrm{OUTPUT}}{\mathrm{INPUT}}$$

When more inputs are used, the equation would be described as following, in which each input and output variable is weighted with a weighting factor (*a_i_* and *b_i_*) (Equation (2)):

(2) $$Ef=\frac{a_{i}Y_{i}}{b_{i}X_{i}}$$

The applied model aims to achieve the maximum amount of output given a certain level of inputs, under a restriction of ignorance of the technological level assumed by each DMU. For this reason, the variable-scale returns model (VRS) proposed by Banker, Charnes, and Cooper [42] is used, oriented toward the output (BBC-output model). Thus, the problem to solve would be the maximization of the following expression (Equation (3)):

(3) $$Max\;y_{j}+\;\varepsilon \left(\sum_{k=1}^{s}h_{k}^{+}+\;\sum_{i=1}^{m}h_{i}^{-}\right)$$

Subject to Equations (4)–(6):

(4) $$\sum_{j=1}^{n}\lambda_{j}*x_{ij}=x_{ij}-h_{i}^{-},\;i=1,\ldots ,m$$

(5) $$\sum_{j=1}^{n}\lambda_{j}*y_{kj}=y_{kj}*\gamma_{j}^{}+h_{k}^{+},\;k=1,\ldots ,m$$

(6) $$\sum_{j=1}^{n}\lambda_{j}=1\;\lambda_{j},\;h_{i}^{-},\;h_{k}^{+}\geq 0,\;\forall i,j,k\;\gamma_{j}^{}free$$

where:

$\gamma_{j}^{}$ is the radial enlargement that occurs in all its outputs. It can be identified with the efficiency of *j* if *j* is compared with a point belonging to the efficient frontier.

$h_{i}^{-}$ is the rectangular reduction of input *i*.

$h_{k}^{+}$ is the rectangular magnification of the output *k*.

$\lambda_{j}$ represents the coefficients of the linear combination of inputs and outputs to which the DMU projection point is referring, on the efficient frontier. It can be interpreted as the proximity of the DMU projection point with respect to the efficient frontier.

In this way, the efficiency frontier would be integrated by all those efficient decision units. Once the border has been determined by these entities, it compares each of the entities that are being studied with the border, under the assumption that the detected deviations indicate inefficient behavior. Thus, one can measure the relative efficiency of a set of DMUs that produce a type of output from a common set of inputs.

Thirdly, a new search was carried out on the main inputs and outputs used by the researchers. The result is shown in Table 1.

Once the bibliographic analysis had been carried out, it was possible to identify the input/output variables that were used in this research, as shown in Table 2.

As can be seen, the investments made in ESF, EAFRD, and ERDF have been taken into account. The resources obtained in the CF and FEMP have not been considered. The reason for this is that due to the very nature of these funds, not all countries in the Eurozone have access to them. In the used DEA model, it is necessary that all the defined variables, both inputs and outputs, have a positive value; thus, it is not admitted variables in which any DMU has a zero value. This parameter would affect the definition of the efficiency frontier, and the value assigned to each of them depends on the closest proximity it has with respect to it.

In any case, the quantified funds (EAFRD, ESF, and ERDF) account for more than 95% of the total distributed funds by the EU, thereby guaranteeing the robustness of the obtained results. This assumption led to not considering countries such as Poland in the analysis. During the period of 2007 to 2013, €67,185,594,244 were allocated to Poland, making it one of the countries that obtained the greatest number of resources from the EU. However, from all of them, almost 40% were cohesion funds. For this reason, and because Poland is not part of the Euro group, it was decided to not consider this country in the analysis.

Three output variables have been chosen. One related to economic growth (GDP per capita) and another two related to the improvement of the environment as a consequence of the use of clean energy: energy production through the use of renewable energies and employment generated in the industry. In this way, it is being measured whether the use of European Funds is allowing sustainable growth by the countries of the Eurozone. If the data indicate that there is no efficiency, it could be interpreted that the bases of economic growth in these countries are produced as a result of the deterioration of the environment due to the use of polluting energies.

**Figure 1:**
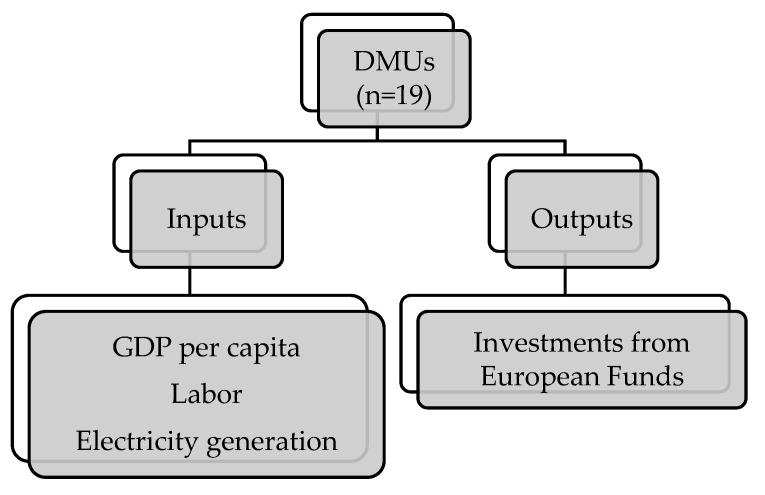
Variables of DEA analysis for environmental efficiency.

GDP per capita, as can be seen in Table 2 is an indicator frequently used by researchers in the DEA analysis applied to the environmental field. The use of the output related to the production of renewable energy is also endorsed by the approval of the European Green Pact in September 2020. In this document, the commission has proposed to achieve a reduction of at least 55% of emissions by 2030 of greenhouse gases. For this, energy efficiency measures and greater use of renewable energies are contemplated. The presentation of the legislative proposals necessary to make this objective a reality is expected to be approved in June 2021. Figure 1 shows a summary of the variables used in DEA analysis.

## 4. Results

Table 3 and Table 4 show the results obtained in the efficiency analyses for the periods of 2007 to 2013 and 2014 to 2018, respectively. In this regard, it should be clarified that the DEA model makes a comparison between the different DMUs (in this article, countries) relating the variable inputs/outputs defined in Section 3. All those DMUs that have a value of 100 are those that are located above the efficiency frontier. The rest shows the relative position of the rest of the countries over them.

As can be seen, there are important differences between the Eurozone countries. Austria, Germany, and Luxembourg have achieved maximum efficiency throughout the period. By contrast, Cyprus, Estonia, Greece, Ireland, Italy, Latvia, Lithuania, Malta, Portugal, Slovakia, Slovenia, and Spain have never achieved that level of efficiency. Thus, although the general average for the period has been, at all times, greater than 50, the Spearman correlation coefficient indicates that the differences in efficiencies between countries are maintained during the period. Therefore, in view of these results, we consider it necessary to articulate mechanisms in the distribution of the funds so that the differences between countries decrease.

In this period, only Malta and the Netherlands have obtained the highest score in the level of efficiency during all the analyzed years. However, the data obtained show how the average of this period is higher than that of the previous period. Therefore, the use of the funds has been more efficient. Probably a legislative development, in this period, the regulation of European resources more respectful to the environment has contributed to this. However, important differences between countries continue to be observed throughout this period, as the Spearman coefficient indicates.

The Spearman rank correlation coefficient during the two analyzed periods has positive values close to 1. This indicates the low mobility of the countries in the position that has been calculated with the DEA analysis. During the period of 2007 to 2013, the average is 0.95, whereas for the following period, the average of the coefficients is 0.90. These scores show that there are countries that, during the two periods, have been highly efficient (such as Austria, Germany, and Luxembourg), whereas others have been very inefficient during the years analyzed (such as Cyprus, Portugal, Slovakia, and Slovenia). Considering these data, it is possible to question the role that the European Funds have been developing as a mechanism for reducing regional differences. These data are corroborated with the calculations made with the basic statistics that are shown in Table 5 and Table 6.

**Figure 2:**
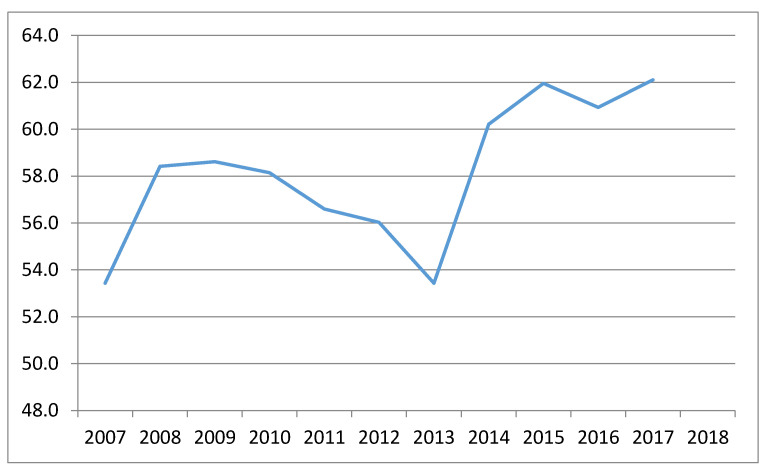
Average evolution of efficiency in the Eurozone countries (period of 2007–2018).

In Figure 2, it can be seen that during the period of 2014 to 2018, the calculated efficiency is higher than that achieved in the period of 2007 to 2013. Specifically, the average of the first period was 56.4, whereas, in the following period, it was 61.3. This represents an increase of 5%. This fact has probably been influenced by the regulatory change of the European Funds. As seen in the introduction section, in the regulations governing the funds for the period of 2014 to 2020, it is observed that sensitivity toward environmental issues was considerably increased, thus reflecting the special concern of European citizens toward this type of issue.

## 5. Conclusions

In the previous analyses of DEA and the environment literature, it was observed that the main variables used by the researchers were related to capital, work, economic growth, and the improvement of a series of environmental indicators. In these studies, comparisons were made between the DMUs analyzed and, based on them, a series of corrective measures that could improve environmental quality that could be determined. Therefore, they moved away from the traditional approaches that related only costs and benefits from an eminently economic perspective.

Based on these analyses, from an academic point of view, a need arose to assess organizations from other perspectives, and, among them, those related to improving the quality of life of people and of the planet.

The results obtained in the previous investigations, and collected in this article, revealed the existence of territorial differences. There is a natural tendency of the economic system to develop regional differences, and these are maintained over time. The differences collected among the different countries of the Eurozone have been maintained throughout the analyzed period. Without the existence of correction mechanisms, such as the European Funds, these results can continue to grow and turn the European space into a territory of inequalities that clashes head-on with the spirit contained in its founding charter.

In the previous literature, important territorial differences have been revealed in eminently economic aspects. We understand that the policies developed by public administrations must also consider, in their objectives, themes related to improving the health of the planet. For this reason, we believe that the main contribution of this research has been to introduce into the DEA model the resources made available in the Eurozone (i.e., the European Funds). The consideration of these resources was taken into account, among many other goals, to unite the idea of growth and respect for the environment. Thus, in this research, a series of output variables related to growth, employment, and energy production in the renewable sector have been defined, and other input variables related to the funds were used to improve growth and employment.

In this research, the goal was to determine the level of efficiency that the Eurozone countries are achieving in the use of European Funds to improve the quality of the environment, mainly in the use of renewable energy.

The European Funds have to meet the goals of improving environmental quality, both directly—approval of specific projects related to the environment—and indirectly—by prioritizing those projects that, without being directly associated with these purposes, contemplate this dimension.

We agree that the European construction must be based on economic growth that makes it possible to bridge the differences between rich and poor regions. It is no less true than in this century. Given the environmental challenges that are being posed, growth should not be a goal in itself if it is not followed by a growth that respects the environment. For this reason, we believe that regional growth projects that pursue the change toward a model in which more renewable energy sources are used should prevail, to the detriment of those who continue to bet on growth based on fossil fuels.

## Figures and Tables

**Table 1:** Input and output variables used by researchers on environmental efficiency.

Authors	Input Variables	Output Variables
bian, Martínez, and Silveira (2012) [29]	Capital Labor Materials Energy	Production Value of service production in each activity CO_2_ emissions (undesirable)
Bian, He, and Xu (2013) [7]	Labor Capital Coal Oil Natural gas Non-fossil energy	Gross Domestic Product (GDP) CO_2_ emissions (undesirable)
Ebrahimi and Salehi (2015) [20]	Human labor Diesel fuel Compost Machinery Chemicals Electricity Water	CO_2_ emission of button mushroom production
Lin and Du (2015) [11]	Capital stock Labor force Energy consumption	Gross domestic product CO_2_ emissions (undesirable)
Song, Hao, and Zhu (2015) [31]	Labor Capital Energy	Added value (desirable) CO_2_ emissions (undesirable)
Duan, Guo, and Xie (2016) [33]	Electricity generation process	Capital Labor Fossil fuel Auxiliary electricity Electricity CO_2_ emissions (undesirable)
Iftikhar, He, and Wang (2016) [34]	Labor Capital Energy	GDP CO_2_ emissions (undesirable)
Tian, Zhao, Mu, Kanianska, and Feng (2016) [36]	Labor Agricultural film Diesel Chemical fertilizers Electricity Pesticides Water Organic fertilizer	Grapes (desirable) Carbon emission (undesirable)
Zha, Zhao, and Bian (2016) [37]	Labor Capital Coal Oil Natural gas	GDP CO_2_
Chen and Geng (2017) [38]	Renewable energy Fossil energy Capital stock Labor force	Real domestic gross product CO_2_ emissions

**Table 2:** Production function of the degree of environmental efficiency.

Type	Variable	Description	
Output	O_ij_: GDP per capita	Annual per capita gross domestic product of the member countries of the Eurozone, where i is the country and j is the year. It is an indicator of the economic situation of a nation. It reflects the total value of all goods and services produced minus the value of goods and services used for intermediate consumption in their production. Expressing GDP in purchasing power standards (PPS) eliminates differences in price levels between countries, and per capita calculations allow comparison of significantly different economies in absolute size. Source: [45]	
	O_ij_: Labor	Employment generated in the field of renewable energies in the member countries of the Eurozone, where i is the country and j is the year. It includes people who work directly or indirectly in the generation of electrical energy based on renewable technologies. Source: [46]	
	O_ij_: Electricity generation from all renewable sources	Electric energy generated using renewable energy as a source from the member countries of the Eurozone, where i is the country and j is the year. Renewable energy is defined as the contribution of renewable energy to the total primary energy supply (STEP). Renewable energies include the primary energy equivalent of hydroelectric (excluding pumped storage), geothermal, solar, wind, tidal, and wave sources. It also includes energy derived from solid biofuels, biogasoline, biodiesel, other liquid biofuels, biogas, and the renewable fraction of municipal waste. This indicator is measured in thousands of toe (oil equivalent tons) and as a percentage of the total primary energy supply. Source: [47] Note: The data corresponding to the years 2007 to 2010 are estimates.	
Inputs	I: Investment in European Funds	I_ij_: FSE	Annual investment of the member countries of the Eurozone in the European Social Fund (ESF), where i is the country and j is the year. Source: [48]
		I_ij_: FEADER	Annual investment of the member countries of the Eurozone in the European Agrigultural Fund for Rural Development (EAFRD), where i is the country and j is the year. Source: [48]
		I_ij_: FEDER	Annual investment of the member countries of the Eurozone in the European Regional Development Funds (ERDF), where i is the country and j is the year. Source: [48]

**Table 3:** Relative efficiency of the Eurozone countries (2007–2013).

Countries/Year	2007	2008	2009	2010	2011	2012	2013
AUSTRIA	100.0	100.0	100.0	100.0	100.0	100.0	100.0
BELGIUM	75.3	77.9	65.0	100.0	100.0	100.0	75.3
CYPRUS	24.6	31.2	31.3	30.4	37.6	27.2	24.6
ESTONIA	17.6	16.5	14.8	14.6	17.9	17.2	17.6
FINLAND	83.9	100.0	99.4	100.0	90.1	81.6	83.9
FRANCE	86.7	95.0	100.0	100.0	96.5	100.0	86.7
GERMANY	100.0	100.0	100.0	100.0	100.0	100.0	100.0
GREECE	24.1	32.2	33.0	29.6	27.1	25.8	24.1
IRELAND	49.8	55.5	52.7	48.8	47.3	48.1	49.8
ITALY	71.3	79.8	85.6	83.2	81.9	75.9	71.3
LATVIA	16.9	24.7	19.9	18.8	14.6	14.9	16.9
LITHUANIA	14.7	14.1	12.4	12.4	13.0	14.2	14.7
LUXEMBOURG	100.0	100.0	100.0	100.0	100.0	100.0	100.0
MALTA	21.9	19.5	20.5	20.8	20.0	21.1	21.9
NETHERLANDS	79.5	100.0	100.0	87.7	83.4	100.0	79.5
PORTUGAL	29.3	34.9	36.5	33.6	31.8	29.8	29.3
SLOVAKIA	19.4	20.2	19.5	18.5	18.5	18.8	19.4
SLOVENIA	24.1	29.2	28.6	26.0	24.5	23.5	24.1
SPAIN	76.1	79.4	94.6	80.5	71.1	66.5	76.1
SPEARMAN COEF.		0.96	0.96	0.93	0.98	0.99	0.95

**Table 4:** Relative efficiency of the countries of the Eurozone (2014–2018).

Countries/Year	2014	2015	2016	2017	2018
AUSTRIA	76.1	74.6	83.4	97.0	92.3
BELGIUM	43.6	42.7	44.1	52.7	28.9
CYPRUS	51.5	51.8	33.1	24.7	6.8
ESTONIA	21.5	24.1	36.5	55.9	60.0
FINLAND	55.1	53.9	55.6	61.0	51.6
FRANCE	100.0	85.8	85.6	83.8	70.7
GERMANY	100.0	100.0	100.0	100.0	84.6
GREECE	24.5	23.8	21.9	22.7	10.2
IRELAND	49.3	63.5	63.1	68.2	11.5
ITALY	91.9	99.6	100.0	91.8	64.0
LATVIA	22.7	34.4	23.2	24.2	6.8
LITHUANIA	21.5	31.3	30.2	24.8	15.0
LUXEMBOURG	100.0	100.0	100.0	100.0	30.4
MALTA	100.0	100.0	100.0	100.0	100.0
NETHERLANDS	100.0	100.0	100.0	100.0	100.0
PORTUGAL	32.6	32.1	28.3	28.4	7.3
SLOVAKIA	21.9	28.1	24.8	21.5	10.3
SLOVENIA	31.7	31.4	28.1	23.3	9.8
SPAIN	100.0	100.0	100.0	100.0	78.8
SPEARMAN COEF.		0.93	0.92	0.95	0.83

**Table 5:** Average efficiency of the countries of the Eurozone (2007–2013).

	Average	No. of Times Maximum Efficiency	Maximum Efficiency	Minimum Efficiency	Variation
AUSTRIA	100	7	100	100	0
BELGIUM	84.7785714	3	100	65.02	34.98
CYPRUS	29.5428571	0	37.59	24.56	13.03
ESTONIA	16.6085714	0	17.94	14.6	3.34
FINLAND	91.2614286	2	100	81.55	18.45
FRANCE	94.9842857	3	100	86.72	13.28
GERMANY	100	7	100	100	0
GREECE	27.9571429	0	32.96	24.06	8.9
IRELAND	50.28	0	55.54	47.25	8.29
ITALY	78.4214286	0	85.56	71.33	14.23
LATVIA	18.0857143	0	24.68	14.6	10.08
LITHUANIA	13.6528571	0	14.74	12.39	2.35
LUXEMBOURG	100	7	100	100	0
MALTA	20.7957143	0	21.87	19.46	2.41
NETHERLANDS	90.0157143	3	100	79.52	20.48
PORTUGAL	32.17	0	36.53	29.27	7.26
SLOVAKIA	19.1885714	0	20.16	18.51	1.65
SLOVENIA	25.7214286	0	29.21	23.46	5.75
SPAIN	77.7485714	0	94.62	66.5	28.12

**Table 6:** Average efficiency of the countries of the Eurozone (2014–2018).

	Average	No. of Times Maximum Efficiency	Maximum Efficiency	Minimum Efficiency	Variation
AUSTRIA	84.684	0	97	74.58	22.42
BELGIUM	42.41	0	52.7	28.86	23.84
CYPRUS	33.582	0	51.83	6.76	45.07
ESTONIA	39.6	0	59.97	21.5	38.47
FINLAND	55.432	0	61	51.55	9.45
FRANCE	85.192	1	100	70.7	29.3
GERMANY	96.918	4	100	84.59	15.41
GREECE	20.598	0	24.45	10.22	14.23
IRELAND	51.112	0	68.19	11.48	56.71
ITALY	89.462	1	100	64	36
LATVIA	22.24	0	34.37	6.8	27.57
LITHUANIA	24.574	0	31.33	15.01	16.32
LUXEMBOURG	86.078	4	100	30.39	69.61
MALTA	100	5	100	100	0
NETHERLANDS	100	5	100	100	0
PORTUGAL	25.732	0	32.55	7.32	25.23
SLOVAKIA	21.316	0	28.1	10.3	17.8
SLOVENIA	24.848	0	31.71	9.75	21.96
SPAIN	95.764	4	100	78.82	21.18

## Data Availability

The data presented in this study are available in Eurostat, EurObserv’ER, and European Commission.
